# Private Lunatic Patients

**Published:** 1903-04-25

**Authors:** 


### HOSPITAL ADMINISTRATION.

### CONSTRUCTION AND ECONOMICS.

### PRIVATE LUNATIC PATIENTS.

The total number of private lunatic patients in England and Wales, registered as such, was on January 1st, 1902, 9,135, and this number was distributed as follows :—In county and borough asylums 1,824, in registered hospitals 3,702, in licensed houses (that is private asylums) 2,891, in naval and military hospitals 254, and 464 were private single patients residing in private houses. It will be seen from the above numbers that one-fifth of the private patients are confined in our county and borough asylums. The distribution of this one-fifth is most unequal. In some county or borough asylums, private patients are not received at all, while in others they constitute an important part of the asylum population. At the City of London Asylum, for instance, there are about 160, at Dorset 100, at Claybury 100, at York 85, at Menston 85, at Exeter 80, at Monmouth 60, at Cumberland 60, and at Worcester 50, while many others contain a few, and a considerable number have none at all. Why this discrepancy? The reason is that very few asylums have followed the example of Claybury and Dorset in providing special accommodation for the lower middle classes of the insane, and that, so far from having spare beds for private patients, they are in a state of chronic arrears with the provision of room for their pauper cases. It is only when a new asylum is opened, or an old one much enlarged, that any considerable number of beds are given up to the use of private patients. The old Lunacy Act did not permit of special buildings being erected at county asylums, nor of private patients being treated differently from the paupers; but these anomalies were removed by the last Act; and yet it is curious to note how rarely this provision has been acted upon, although it would be beneficial alike to the ratepayer and the private patient. We strongly hold the opinion that annexes should be put up at all our county and borough asylums, unless such asylums are already of unwieldy bulk. At the same time we have no wish to see our public asylums entering into competition with private asylums; and hence the sums charged for maintenance should not greatly exceed the ordinary maintenance rate plus the county rate and a reasonable addition for interest on capital. Probably an average of one guinea a week would more than cover all these: and the class provided for should be just above that found in county asylums nominally rated as paupers. The accommodation for this class is at present extremely deficient, and too often the difficulty is got over by calling in the relieving officer, and through him the patient is sent to the county asylum as a pauper although the guardians may subsequently call on the relatives to refund the whole of the asylum charge. Thus there may be no actual loss to the county, nor is there any gain; but the spirit of the Act is broken or at least stretched.

When we come to the lower fringe, or rather to the less well-off section of the professional classes, we find that the same difficulty of finding accommodation is present, often in an aggravated form, because to be sent to a county asylum is in the highest degree repugnant; and that the lunatic hospitals originally intended for this very class, and perhaps the class already referred to, are being more and more given up to richer people who could well afford the charges in our best private asylums, and so it has come to pass that the less fortunate brethren are driven to the county asylum, or, as sometimes happens, they are received at our private asylums at barely remunerative charges. And it is not the mere presence of a few of these patients in our lunatic hospitals which constitutes the evil. It is that such "good" patients are indulged with as much accommodation and attendance as would suffice for two, three, or, may be, four, of the poorer professional class we speak of. The cost per head rises greatly with the reception of these rich patients; and if it should happen that a few patients are admitted at anything less than the average rate, we are reminded of the "charitable" work done by the hospital, although the average sum paid by these "charity" cases may be as much as they could be received for in some of our private asylums. Where, then, does the charity come in? In saying this we are not unmindful that some of these hospitals—Oxford, Lincoln, and others—do their best to admit those deserving cases; but many of them are crippled by the smaller sizes of the hospitals. It is unfortunately too true that charitable bequests often stop short of the insane. A general infirmary will raise twenty thousand pounds as easily as a lunatic hospital will raise as many hundreds; and it may well be that would-be donors are prevented from giving by the fear that their money might be used for providing accommodation for patients paying three or four guineas a week. The medical man who wishes to obtain admission for a patient will often find that no vacancy exists except for those who can afford the higher rates; and the difficulty is increased if the patient should be epileptic or paralytic.

We have already stated that the private asylums contain 2,891 patients, which shows that the friends of 2,891 insane persons believe in the administration of these asylums; and we have no hesitation in saying that in the overwhelming majority of instances this confidence is not misplaced. Here then is the justification for their existence. Regarding the smaller ones it is certain that no lunatic hospital can compare with them in their home-like appearance, and in the family lives which the patients lead. We stated in a former article that while these smaller asylums should not have additions made to them, there were others which could most advantageously have their licenses increased. As a matter of fact, the private patients increased by 118 during the year 1901, and yet under the existing law no enlargement of private asylums is possible.

There is, however, one point connected with our private asylums which the public never seems to realise, and that is that some half-dozen of them are allowed to receive pauper patients as well as private patients. On the date of the last issued Blue Book there were 1,172 paupers in these asylums. We see no reason for this, and if it were prohibited two good things would result. Firstly, the vacant beds would be gradually filled by private patients paying low rates for maintenance, as it is almost exclusively the very large private asylums which receive both classes; and, secondly, there would be a saving of something like £20,000 a year to the ratepayers.

The number of private single patients residing in private houses, and especially in the houses of medical men, should be susceptible of large increase. There are many patients ever on the borderland of insanity. Occasionally they step over the border, and can be then certified; but often they do not come clearly within the Act, and yet they urgently need medical care and supervision. Medical men are shy of receiving these cases, as they dread having to defend an action for contravening the Lunacy Law, and it must happen often that the patient drifts into incurable insanity, which might have been prevented under a better state of things. Future legislation might deal with this problem.

To recapitulate:—1. County and borough asylums should build annexes of sufficient size to receive all patients of the lower middle class in their respective districts. 2. The lunatic hospitals should, as far as possible, be restricted to the admission of charity cases. 3. The smaller private asylums should be kept as they are. 4. Some of the best managed of the moderate-sized private asylums should have the number they are licensed for considerably increased. 5. Those private asylums, at present allowed to take in paupers, should have their licenses altered to prevent this in future. 6. The number of private single patients ought to be greatly increased.

